# Multifactorial Microvariability of the Italian Raw Milk Cheese Microbiota and Implication for Current Regulatory Scheme

**DOI:** 10.1128/msystems.01068-22

**Published:** 2023-01-23

**Authors:** Federico Fontana, Giulia Longhi, Giulia Alessandri, Gabriele Andrea Lugli, Leonardo Mancabelli, Chiara Tarracchini, Alice Viappiani, Rosaria Anzalone, Marco Ventura, Francesca Turroni, Christian Milani

**Affiliations:** a Laboratory of Probiogenomics, Department of Chemistry, Life Sciences, and Environmental Sustainability, University of Parma, Parma, Italy; b GenProbio srl, Parma, Italy; c Microbiome Research Hub, University of Parma, Parma, Italy; Teagasc Food Research Centre

**Keywords:** raw milk cheese, microbiota, metagenomics

## Abstract

Raw milk cheese manufactory is strictly regulated in Europe by the Protected Designation of Origin (PDO) quality scheme, which protects indigenous food products based on geographical and biotechnological features. This study encompassed the collection of 128 raw milk cheese samples across Italy to investigate the resident microbiome correlated to current PDO specifications. Shotgun metagenomic approaches highlighted how the microbial communities are primarily linked to each cheesemaking site and consequently to the use of site-specific Natural Whey Cultures (NWCs), defined by a multifactorial set of local environmental factors rather than solely by cheese type or geographical origin that guide the current PDO specification. Moreover, in-depth functional characterization of Cheese Community State Types (CCSTs) and comparative genomics efforts, including metagenomically assembled genomes (MAGs) of the dominant microbial taxa, revealed NWCs-related unique enzymatic profiles impacting the organoleptic features of the produced cheeses and availability of bioactive compounds to consumers, with putative health implications. Thus, these results highlighted the need for a profound rethinking of the current PDO designation with a focus on the production site-specific microbial metabolism to understand and guarantee the organoleptic features of the final product recognized as PDO.

**IMPORTANCE** The Protected Designation of Origin (PDO) guarantees the traceability of food production processes, and that the production takes place in a well-defined restricted geographical area. Nevertheless, the organoleptic qualities of the same dairy products, i.e., cheeses under the same PDO denomination, differ between manufacturers. The final product’s flavor and qualitative aspects can be related to the resident microbial population, not considered by the PDO denomination. Here, we analyzed a complete set of different Italian cheeses produced from raw milk through shotgun sequencing in order to study the variability of the different microbial profiles resident in Italian PDO cheeses. Furthermore, an in-depth functional analysis, along with a comparative genomic analysis, was performed in order to correlate the taxonomic information with the organoleptic properties of the final product. This analysis made it possible to highlight how the PDO denomination should be revisited to understand the effect that Natural Whey Cultures (NWCs), used in the traditional production of raw milk cheese and unique to each manufacturer, impacts on the organoleptic features of the final product.

## INTRODUCTION

According to the European Food Safety Authority (EFSA), raw milk is defined as milk produced by farm animals, generally cows, sheep, goats, and buffaloes, which has neither been heated above 40°C nor subjected to any other treatment having an equivalent effect on the milk-associated microbial community (1). Therefore, while direct consumption of raw milk can expose to microbiological hazards (2), the presence of endogenous living microorganisms is considered responsible for the complex and interesting organoleptic features of raw milk cheeses compared to those derived from pasteurized milk (3). In this context, raw milk cheesemaking is strictly regulated in Europe by the Protected Designation of Origin (PDO) product quality scheme, which links products to their geographic origins by ensuring production, processing, and preparation within a specific geographical area that follows specific regulated procedures, employing expertise of local producers and raw materials from the geographical environment concerned.

In the case of raw milk cheeses, the key factor defining the resident microbial community is the use of back-slopping, which consists of using natural whey cultures (NWCs) as bacterial starters instead of commercially available strains. NWCs consist of fermented milk harboring a complex microbial community from the raw milk that is constantly added at each production cycle (4), similarly to the use and maintenance of sourdough in breadmaking. Due to its nature, NWCs are extremely variable in relation to each specific production site and modulated by local environmental factors (5).

In this context, the structural and physical-chemical modifications induced during fermentation of the milk matrix by the indigenous microbial communities originating from NWCs are the fundamental biochemical process responsible for the texture and other functional qualities of dairy products (6–9). Indeed, the organoleptic characteristics of fermented dairy products, such as texture, aroma, and flavor depend on the profile of molecules released by the microbiome-driven chemical conversion of carbohydrates, lipids, fats, and proteins, typically contained in milk (10–16). Moreover, the profile of functional molecules released by the local microbiota during cheese ripening will be metabolized by the human cheese consumers, thus exerting relevant biological roles impacting systemically on the human health and well-being. Yet, despite this marked relevance of the microbial metabolism in cheesemaking, the cheese microbiomes and their productions site-specific high variability are only marginally considered in the current PDO regulations.

Due to the importance of the cheese microbiota in cheesemaking, many efforts have been made to understand the taxonomic composition and functional role of the microbial communities found in Italian cheeses (17–19). Nevertheless, a comprehensive dissection of the genomic and functional biodiversity of the microbiota harbored by PDO raw milk cheeses produced across the Italian peninsula is still missing. For this reason, we sampled 128 PDO raw milk cheeses covering all the main Italian types of cheese products (20), whose microbial populations and corresponding metabolic potential have been assessed through shotgun metagenomics using both short- and long-read sequencing approaches.

## RESULTS AND DISCUSSION

### Metagenomic characterization of the bacterial community of PDO Italian raw milk cheeses.

**FIG 1:**
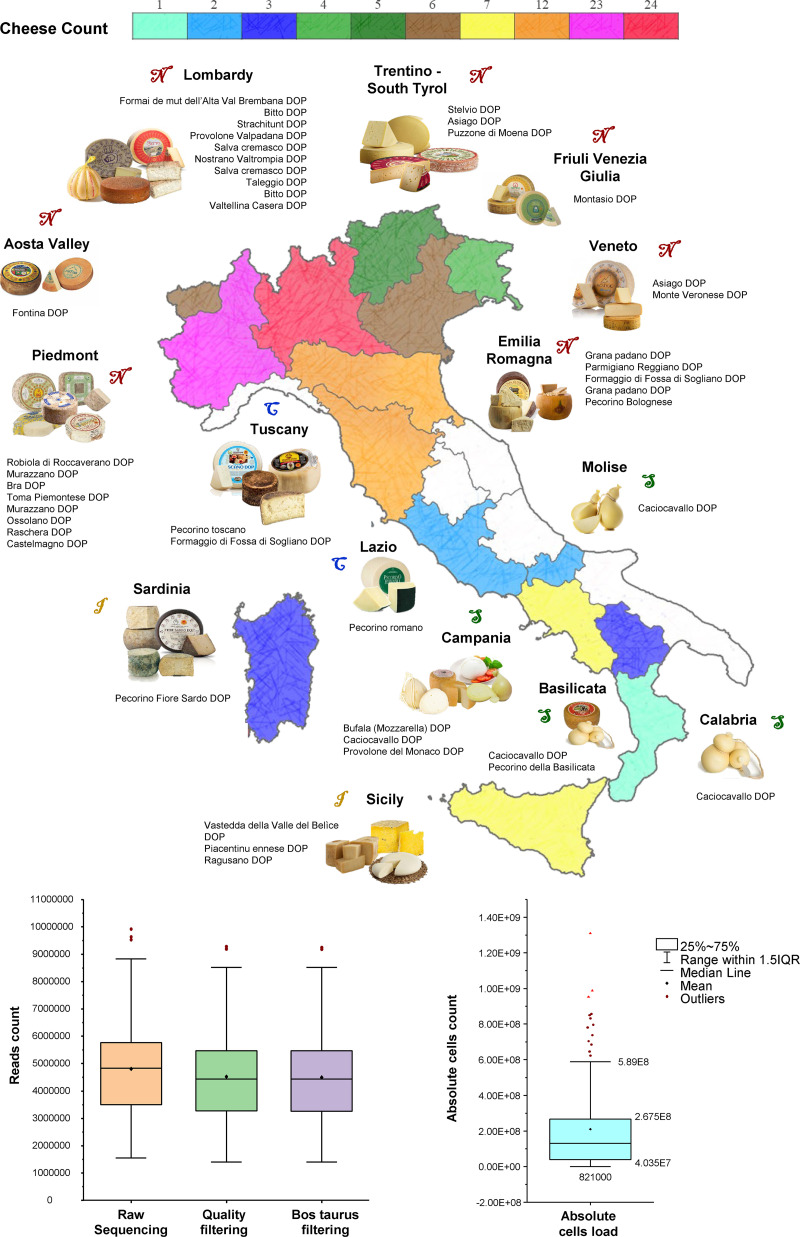
Geographical distribution of collected cheeses. (a) Schematic representation of Italy, with regions colored according to the number of cheeses collected. For white regions, samples of cheeses have not been collected. Pictures of main cheeses from each region are reported. (b) Whisker plot representing the sequencing depth from raw to filtered reads, while (c) is a Whisker plot representing the absolute cells count distribution of each cheese.

In the framework of this study, we collected up to five samples for each of the main PDO raw milk cheeses produced in Italy (Fig. 1). These are artisanal raw milk cheeses produced following the PDO guidelines and employing a cheesemaking technique named back-slopping, in which a small portion of the previous batch of fermented milk is used to support the next fermentation step of raw milk without adding commercial bacterial starters (4). This approach consists of a preactivated microbial starter, selected during multiple back-slopping cycles, and thus, historically unique to each cheesemaking site. Furthermore, as this microbial starter is kept in continuous growth thanks to the daily addition of fresh raw milk, it also adapts to local variables on a microgeographical scale such as temperature and humidity levels, ultimately causing fluctuations in the final organoleptic features of the dairy product.

Overall, we retrieved a total of 103 cheese samples corresponding to 32 PDO cheese types collected across the Italian peninsula, including multiple cheesemakers for cheese type (Fig. 1) (Data Set S1). Furthermore, for comparison purposes, we also collected 25 samples of non-PDO cheeses, i.e., an (unpasteurized) raw-milk cheese type without PDO certification, which were manufactured with the artificial addition of selected microbial starters. Microbial DNA extracted from the collected samples was submitted to shotgun sequencing and raw reads were processed through the METAnnotatorX2 pipeline (21) in order to obtain species-level taxonomic profiles (Data Set S1) (Fig. 1). Subsequently, a flow cytometry assay of the total bacterial load present in 0.2 g of cheese was used to transform the relative abundance of each profiled microbial taxa into absolute abundance, i.e., estimation of species-specific cells load (Data Set S1) (Fig. 1). Notably, no correlation was found between alpha diversity expressed as the number of observed species and PDO designation (Independent T-test *P*-value >0.05) (Data Set S1).

### Multifactorial dissection of the species-level taxonomic composition across PDO and non-PDO Italian raw milk cheeses.

The species-level taxonomic composition of each cheese profile used in this study was explored to evaluate its variability across the Italian peninsula, considering both PDO and non-PDO cheeses. Intriguingly, prevalence analysis of bacterial species showed that 11 taxa could be found in at least 10% of the Italian PDO cheeses, corresponding to Streptococcus thermophilus (prevalence of 81.5%), six *Lactobacillus* species (prevalence ranging from 12.6% to 60.9%), Lactococcus lactis (prevalence of 42.7%), *Lactiplantibacillus plantarum* (prevalence of 17.5%), Leuconostoc mesenteroides (prevalence of 12.6%), and Bifidobacterium mongoliense (prevalence of 11.6%) (Data Set S1).

Notably, despite a core microbiota consisting of 11 highly prevalent species, visualization of the intersample’s taxonomic diversity (beta-diversity) through a two-dimensional principal coordinate analysis (PCoA) revealed the absence of evident clustering of cheeses based on cheese type or regional localization (Fig. S1). Nevertheless, validation through ANOSIM analysis revealed an R correlation of 12.8% (*P* < 0.005) (Fig. S1) indicative that geographical region partially participate in defining the taxonomic composition. In-depth statistical investigation (detailed in the Text S1) ultimately revealed that this result is due to the specific use of Lactococcus lactis as microbial starter in non-PDO cheeses from Tuscany, specifically Pecorino Toscano (Data Set S1). In contrast, no correlation between geographical region and cheese microbiota was found for PDO cheeses (Data Set S1).

10.1128/msystems.01068-22.10 TEXT S1 Supplementary text contains additional detailed analysis regarding the taxonomical compositions between PDO and non PDO cheeses with the respective statistical confirmation, followed by a detailed bivariate correlation analysis between the bacterial species found inside the analyzed pool of PDO cheese. Download TEXT S1, DOCX file, 0.02 MB. Copyright © 2023 Fontana et al. 2023 Fontana et al. https://creativecommons.org/licenses/by/4.0/ This content is distributed under the terms of the Creative Commons Attribution 4.0 International license.

10.1128/msystems.01068-22.11 DATA SET S1 Metadata, Metadata summary, Taxonomical profiles (relative abundance), Cytofluorimetry-normalized taxonomical profiles (absolute abundance), Geographical based ANOVA analysis of Taxonomical data, Enzymatic profiles (EC numbers with prevalence > 5%), Alpha diversity and Absolute cells count, PDO prevalence heatmap, CCSTs composition in absolute cell count, Correlation between matrix type and HPCCSTs, Species correlation analysis, functional and flavor EC analysis subdivided for HPCCSTs, Pangenome - PGAP Analysis, *L. paracasei* unique genes analysis, *L. delbrueckii* unique genes analysis, *S. thermophilus* unique genes analysis. Download DATA SET S1, XLSX file, 4.7 MB. Copyright © 2023 Fontana et al. 2023 Fontana et al. https://creativecommons.org/licenses/by/4.0/ This content is distributed under the terms of the Creative Commons Attribution 4.0 International license.

10.1128/msystems.01068-22.1 FIG S1 Bidimensional PCoA representation of samples subdivided for cheese type denomination (based on PDO specification) and for region of production. In addition, an ANOSIM analysis regarding regional subdivision is reported at the bottom of the figure. Download FIG S1, TIF file, 1.8 MB. Copyright © 2023 Fontana et al. 2023 Fontana et al. https://creativecommons.org/licenses/by/4.0/ This content is distributed under the terms of the Creative Commons Attribution 4.0 International license.

To carry out a comprehensive and complete analysis, cheese matrix hardness was also evaluated as another high-relevant metadata, related directly to the ripening time, which may impact the cheese microbiota’s taxonomic composition (18, 22). Therefore, each cheese sample was categorized as hard, semi-hard, and soft cheese. This investigation highlighted that there is a correlation between matrix type and microbial composition (ANOSIM R 15.6%, *P* < 0.001) (Fig. S3). Then, through a PCoA analysis, we noticed that most cheeses with hard matrices tend to cluster together. In contrast, semihard and soft cheeses did not show any particular clustering profile (Fig. S3). In detail, between the hard cheeses only two PDO types seem to cluster together, i.e., Parmigiano Reggiano And Grana Padano (Fig. S1, Fig. S2 and S3). These two cheese types are hard and long-aged dairy products, which is a factor that leads to a decrease in the organic substrate initially present in the fresh, nonaged cheese matrix. As a result of this modification, a simplification of the resident microbiota occurs (average species richness of 6.5), which is reflected in the reduction of dispersion observed in the beta diversity analysis (Data Set S1) (Fig. S1 and S3).

10.1128/msystems.01068-22.2 FIG S2 Bidimensional PCoA representation of samples subdivided for PDO and non-PDO category, with related PERMANOVA and ANOSIM analysis at the bottom of the figure. Download FIG S2, TIF file, 1.6 MB. Copyright © 2023 Fontana et al. 2023 Fontana et al. https://creativecommons.org/licenses/by/4.0/ This content is distributed under the terms of the Creative Commons Attribution 4.0 International license.

10.1128/msystems.01068-22.3 FIG S3 Bidimensional PCoA representation of samples subdivided for cheese matrix hardness, with related ANOSIM analysis. Download FIG S3, TIF file, 1.6 MB. Copyright © 2023 Fontana et al. 2023 Fontana et al. https://creativecommons.org/licenses/by/4.0/ This content is distributed under the terms of the Creative Commons Attribution 4.0 International license.

These observations highlight how the microbial particularities of the different cheese products with the same ripening stage are multifactorial and linked to the dairy site as a unique and comprehensive sum of each impacting factor while cheese aging will eventually induce a simplification of the microbial population. Nonetheless, further investigations are required to validate this approach, with particular focus on direct NWCs compositions and their seasonal composition stability.

### Ecological investigation of co-occurrent microbial communities in Italian raw milk cheeses.

After evaluating the main metadata that could impact on the composition and stability of the cheese microbiota, the relative abundances of microbial profiles were normalized using the absolute cell load obtained from flow cytometry assays (Data Set S1) (Fig. 1).

**FIG 2:**
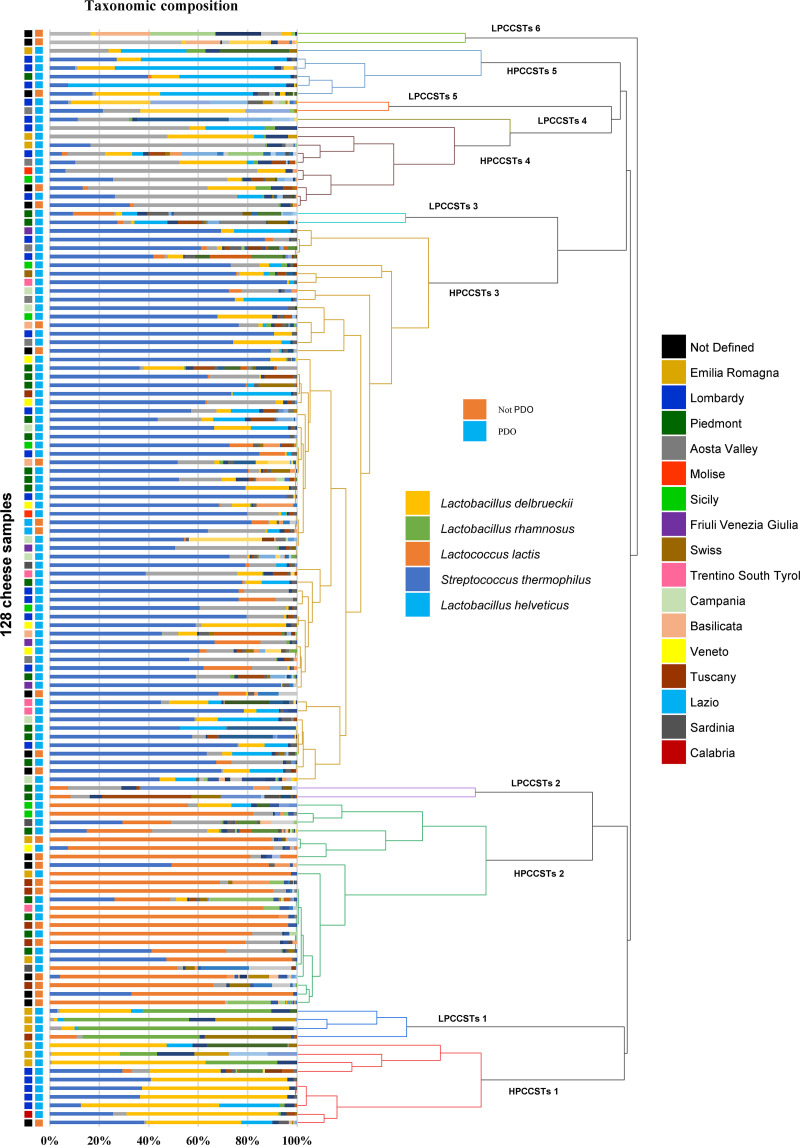
HCL subdivision of all cheese samples. Graphic representation of HCL subdivision of cheese samples is reported, with branch colored based on HCA cluster. In addition, a stylized taxonomic profile of samples is shown along with PDO/non-PDO classification, geographical designation and legend of the main taxa are reported.

Then, to define microbial characteristics shared by different clusters of cheese samples, a hierarchical clustering analysis (HCA) was performed based on their absolute abundance composition, leading to the definition of five high prevalence cheese community state types (HPCCSTs), i.e., high prevalence recurring microbial profiles, found in at least five among the 128 Italian raw milk cheeses collected in this study (Fig. 2) (Data Set S1). The average bacterial load observed for the predicted HPCCSTs ranged from 6.14E + 07 to 2.44E + 08 (Data Set S1).

**FIG 3:**
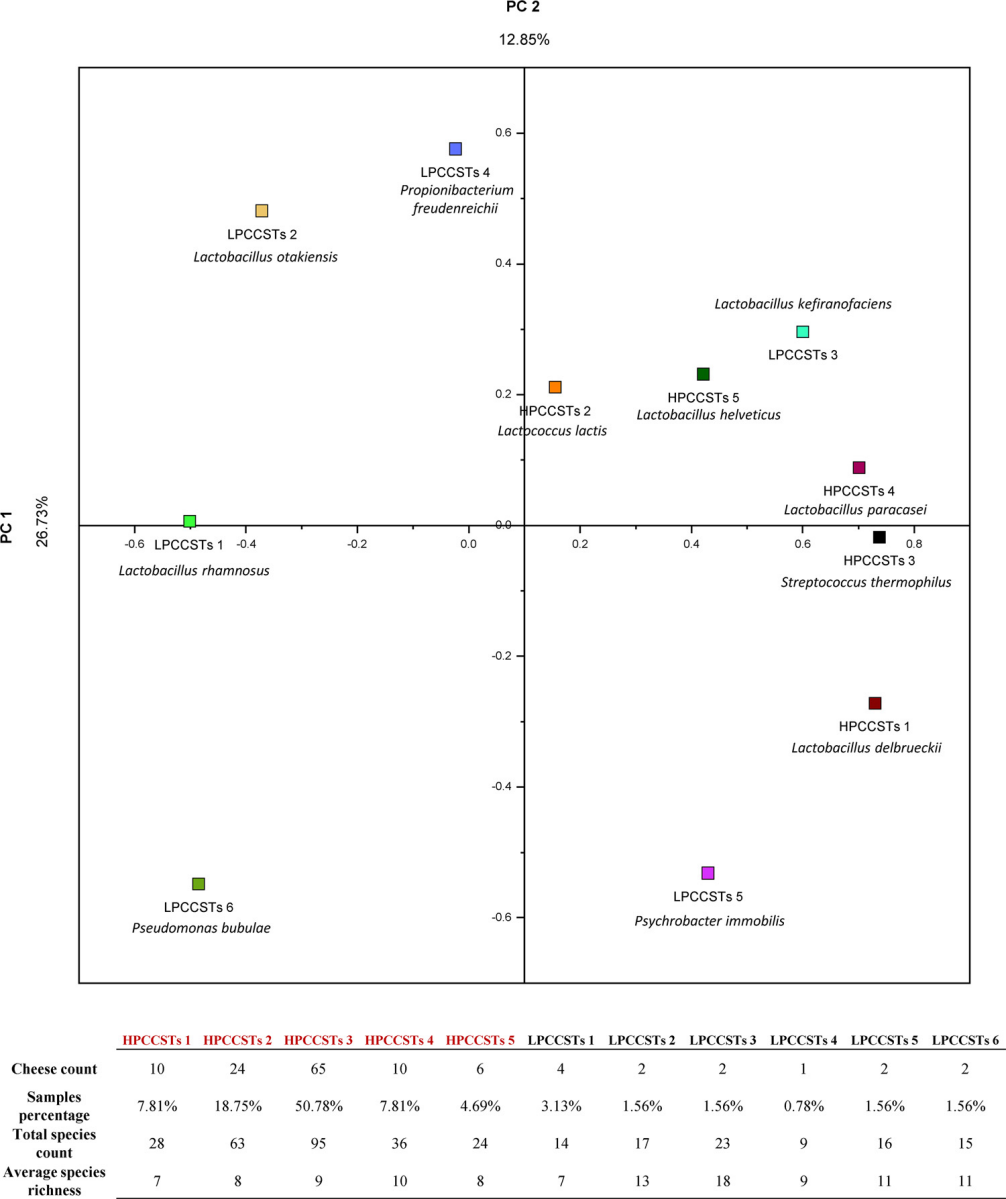
PCoA of CCSTs Bray Curtis dissimilarity matrix. PCoA representation of beta diversity among the different CCSTs acts as a centroid for all the samples belonging to each CCST. Each CCST showed an average absolute composition based on the samples’ absolute cell composition. Furthermore, the beta diversity score was based on a Bray-Curtis dissimilarity matrix to collapse the weight of each bacterial species into a single microbiological distance value to normalize the results and highlight the macro differences in microbial composition among the various CCSTs. Finally, near each CCSTs square point is also reported the predominant bacterial species for each CCST, as well as a summary of the main data regarding CCSTs species richness and sample count.

The five HPCCSTs are characterized by an average species richness ranging from seven to 10, with five species acting as (co)dominant by constituting on average >57% of the HPCCSTs’ microbial community along with the relevant participation of accessory taxa. In detail, S. thermophilus resulted dominant in HPCCST 3 and co-dominant in all the other four HPCCSTs, as expected by a thermophilic lactic acid bacteria (LAB) (23). Instead, *Lactobacillus* species *L. delbrueckii*, *L. paracasei*, and L. helveticus as well as Lactococcus lactis act as dominant bacterial species in HPCCST 1, HPCCST 4, HPCCST 5 and HPCCST 2, respectively (Fig. 2 and 3; Fig. S4) (Data Set S1).

10.1128/msystems.01068-22.4 FIG S4 (a) Bidimensional PCoA representation of samples subdivided for HPCCSTs clusterization is reported. (b) Two bar plots, one showing the average relative abundance composition of each HPCCSTs and the other the absolute compositions of each HPCCSTs based on the total cell load normalization. (c) Average species richness for each different HPCCSTs. Download FIG S4, TIF file, 1.9 MB. Copyright © 2023 Fontana et al. 2023 Fontana et al. https://creativecommons.org/licenses/by/4.0/ This content is distributed under the terms of the Creative Commons Attribution 4.0 International license.

Furthermore, the HLC analysis also revealed six low prevalence CCSTs (LPCCSTs) supported each by less than five cheese samples (Fig. 2 and 3; Fig. S4) (Data Set S1). In detail, LPCCSTs 5 and 6 represent clusters of contaminants that can be typically found in dairy production (Fig. 2 and 3) (Data Set S1) (24, 25).

As expected, evaluation of the distribution of non-PDO cheeses showed that they fall mainly in HPCCSTs 2 and 3 dominated by L. lactis and S. thermophilus, which are among the most common species exploited as artificial microbial starters in cheese manufacturing (26, 27) (Fig. S5) (Data Set S1). Subsequent statistical analyses were performed considering only PDO cheeses falling in the predicted CCSTs. Notably, we could not identify any clear correlations between cheese types or geographical origins and specific HPCCSTs, remarking that each production site has a major role in defining the cheese microbiota (Fig. S5). In addition, when the type of cheese matrix type (soft, semi-hard, and hard) was correlated with the predicted HPCCSTs, it resulted that only semi-hard cheeses weakly and positively correlate (cor. 0.2037) with HPCCST 3 (*P* < 0.05) (Data Set S1).

10.1128/msystems.01068-22.5 FIG S5 (a) Bar plot showing the PDO/non PDO composition for each CCSTs. (b) Bar plot showing the regional subdivision of samples inside each CCSTs. (c) Bar plot showing the cheese type subdivision of samples for each CCSTs. Download FIG S5, TIF file, 2.3 MB. Copyright © 2023 Fontana et al. 2023 Fontana et al. https://creativecommons.org/licenses/by/4.0/ This content is distributed under the terms of the Creative Commons Attribution 4.0 International license.

These data confirm that cheese type-specific cheesemaking practices and cheese-related features like diary-matrix hardness have limited impact on the final microbial population harbored by the Italian raw milk cheeses collected. Instead, we propose that the microgeographical uniqueness of each cheesemaking site over the cheese-type denomination represents the main driving force, with a putative key role of NWCs modulated by their unique local environmental factors (moisture, temperature, etc.), along with the microbiota that naturally harbor in the local raw milk.

In the framework of this study, we also investigated the relationship between the bacterial species resident in PDO cheeses and the HCPPSTs through a bivariate correlation analysis that allowed the dissection of their ecological relationships (additional exhaustive discussion can be found in Text S1).

### Reconstruction of the metabolic potential of PDO Italian raw milk cheese’s microbiota involved in developing cheese’s organoleptic features.

**FIG 4:**
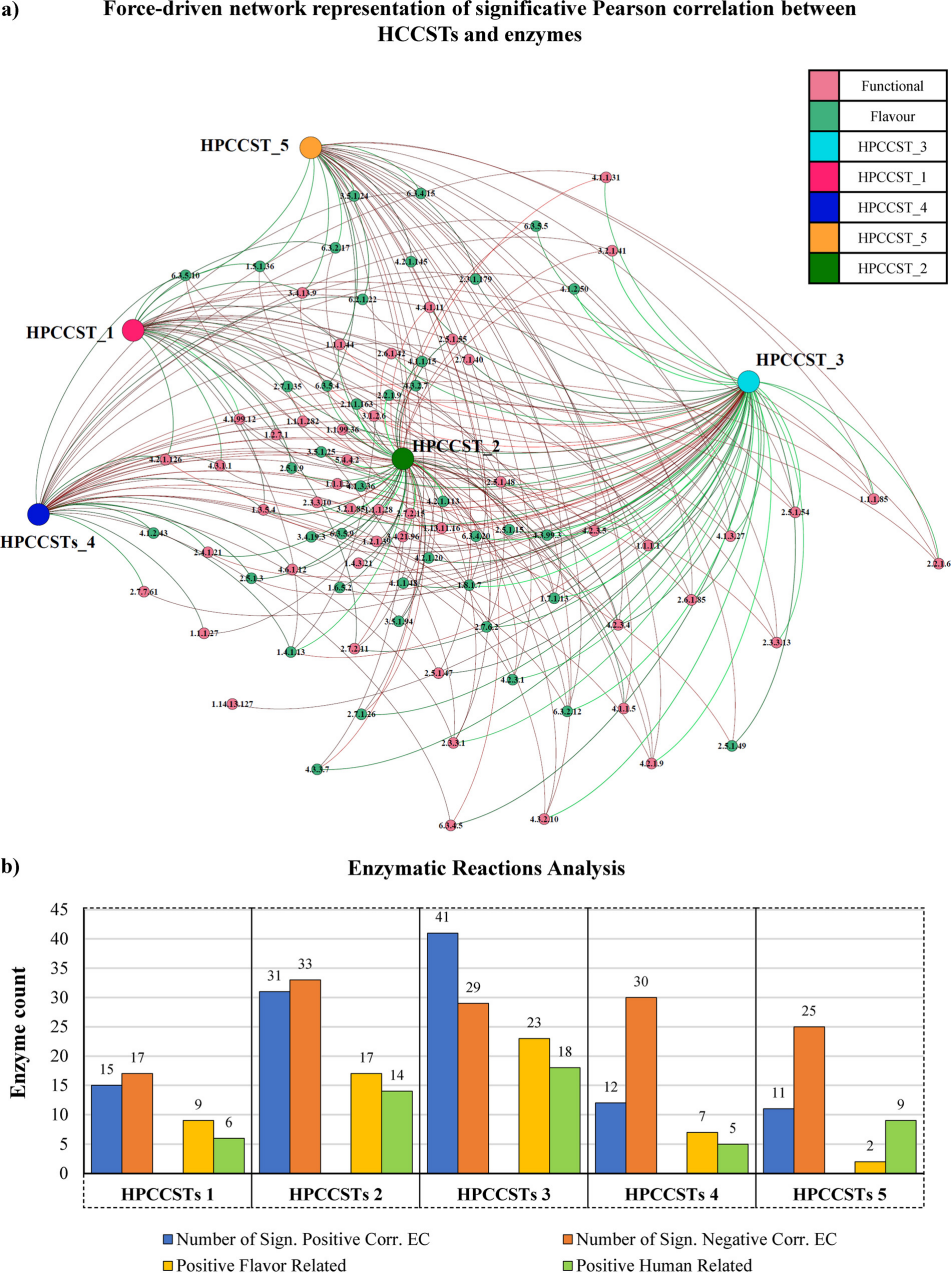
Human and flavor EC reports. (a) Network representation of correlation analysis based on a significative statistical relationship between the EC – numbers (enzymes) and HPCCSTs. Additionally, nodes were colored in order to separate flavor (green) and human health-related (pink) enzymes. (b) Bar-plot graph showing correlations data regarding human health-supporting and flavor enzyme count and HPCCSTs. In detail, the blue bar represents the sum of all positive correlations between CCST and EC, the orange bar represents the sum of all negative correlations between CCST and EC, the yellow bar represents the sum of all positive correlations with EC numbers relating to the flavor enhancement and the green bar represents the sum of all positive correlations with EC numbers relating to human health-supporting functions (vitamin precursor etc.).

After identifying the most common taxonomic profiles, also known as CCSTs, and how their species correlate, we evaluated how these different taxonomic clusters can organoleptically influence the final cheese product through their microbial metabolism. Thus, shotgun metagenomics data of PDO cheeses were submitted to functional metabolic profiling by METAnnotatorX2 to evaluate the commitment of each HPCCSTs toward a manually curated database of enzymatic reactions. This process allowed to reconstruct a functional profile covering a total of 1,746 enzymatic reactions that showed >5% prevalence between the pool of 128 cheese samples analyzed. Because the data used are based on shotgun metagenomics with high-depth sequencing, this functional analysis was able to trace genes present in extremely low number of copies in the whole metagenome (<0.000002% in relative abundance). Then, following a Pearson correlation analysis, we extracted a subset of 48 statistically significant enzymatic reactions (28) that participate in the establishment of the cheese’s organoleptic features and correlate with at least one of the HPCCSTs (26–31) (Data Set S1) (Fig. 4). The selection of these 48 enzymatic reactions from the correlation pool was performed manually, exploiting what is reported in the recent literature (32–35) and selecting relevant enzymes along with products and by-products of organoleptic interest. In detail, selected enzymatic reactions refer to flavor enhancer molecules like acetaldehyde, ethanol, lactate, and acetoin, other than technical agents like LPS-related enzymes (enhancer of texture in yogurt and other fermented dairy products) (Data Set S1). Additional information concerning the selected enzymes and their correlation score with the HPCCSTs are available in the Data Set S1.

**FIG 5:**
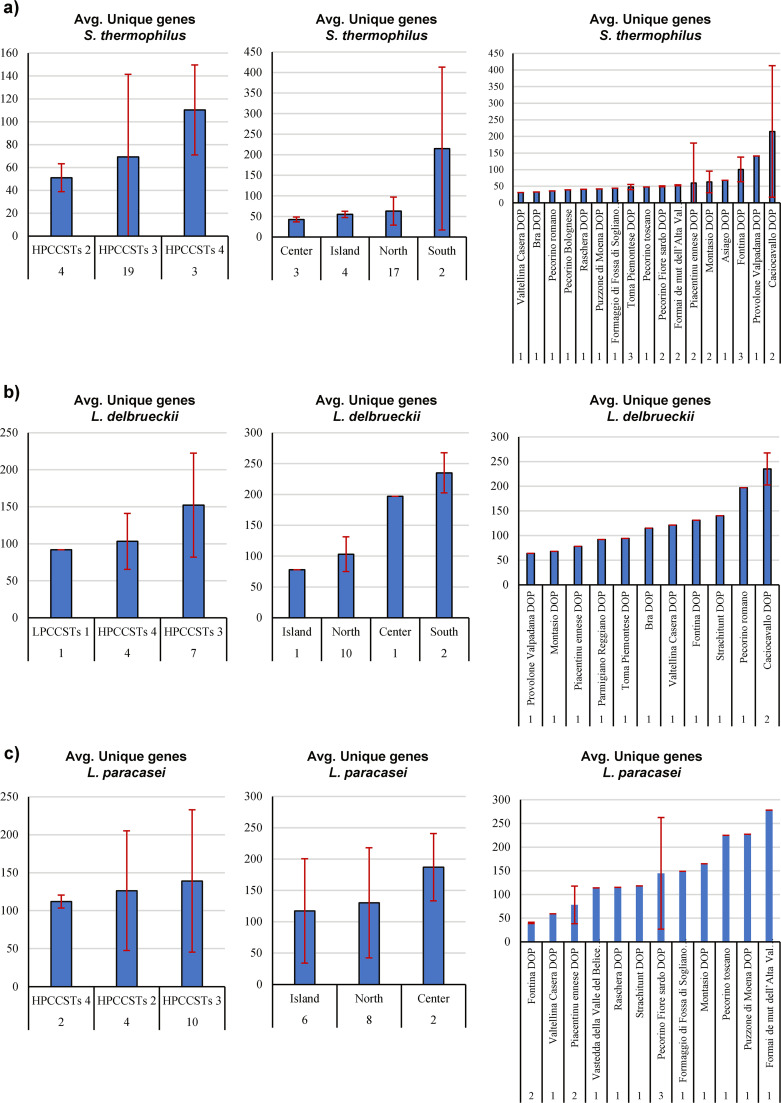
Comparative genomics analysis on unique genes content and metadata subdivision. (a) Three panels, showing the average unique genes content between S. thermophilus strains inside HPCCSTs clusters (first panel), between macro geographical area (second panel) and between cheese types (third panel), with the standard deviation reported when possible. (b) Three panels showing the average unique genes content between *L. delbueckii* strains inside HPCCSTs clusters (first panel), between macro geographical area (second panel) and between cheese types (third panel), with the standard deviation reported when possible. (c) Three panels showing the average unique genes content between *L. paracasei* strains inside HPCCSTs clusters (first panel), between macro geographical area (second panel) and between cheese types (third panel), with the standard deviation reported when possible.

In detail, the number of positive correlations with enzymes inherent to organoleptically relevant flavors ranged from 2 (HPCCST_5) to 23 (HPCCST_3) (*P* value < 0.001) (Fig. 5). Notably, this result may represent the foundation of the differences in the organoleptic features observed for the same raw milk cheese type produced by different cheesemakers, as also suggested by the distribution of CCSTs across the collected types of cheese described above (Data Set S1). Thus, emphasizing the key role in organoleptic features development exerted by specific microbial consortia. Specifically, once the microbiological profile has been categorized into one of the HPCCSTs categories, it is possible to trace a specific and expected metabolic potential in the final product, thus increasing our understanding of the possible organoleptic and health implications. Nonetheless, this needs to be confirmed through future RNA profiling and metabolomics studies regarding the actual expression of these 48 enzymes.

Subsequently, the average relative abundance of functional enzyme-encoding reads for each HPCCSTs analyzed was normalized using the absolute cell load obtained from flow cytometry data (Data Set S1) (Fig. 1). This normalization of the functional profiles for the average bacterial load evidenced that the differences in average bacterial load observed for the predicted HPCCSTs (ranging from 6.14E + 07 to 2.44E + 08) may markedly impact their resulting metabolic activity (Data Set S1).

These data remark that the metabolic potential of the resident microbial population is probably linked to the manufacture-specific uniqueness (NWC and other environmental factors) (Data Set S1). Altogether, these results strengthen the notion that dissection of CCSTs composition and metabolic potential, coupled with bacterial load assessment, is a valuable target for food fingerprinting aimed at PDO cheese overall enhancement of the organoleptic and health-related features.

### Predicted metabolites of raw milk cheese microbiota with potential impact on human physiology.

Recently, it has been demonstrated that the microbial community harbored by raw milk cheeses can colonize the gut of human consumers, where it can persist for weeks, especially when supported by a diet rich in milk and its derivates (36). Moreover, lactic acid bacteria (LAB) can also accumulate important secondary metabolites into cheese products, making them a natural supplement of important fermentation by-products (27, 31). For this reason, functional profiling of the cheeses’ microbiota was employed to perform an explorative analysis of how each HPCCSTs-related enzymes may impact consumers’ health. Therefore, a subset of 40 enzymatic reactions which showed statistically relevant correlation and that lead to the production of high-interest microbial metabolites (37) was extracted (Data Set S1) (Fig. 4).

In detail, among the 40 enzymes, selected manually based on recent scientific literature, there are enzymes participating in pathways that can lead to the production of vitamins or their precursors, such as the folate pathway (EC 2.5.1.15, related to vitamin B9), the menaquinone-biosynthesis pathways (EC 2.1.1.163, related to vitamin K2), flavin (EC 1.5.1.36, related to vitamin B2), and a precursor of vitamin B12, adenosylcobyrate (EC 6.3.5.10) (38–40). Furthermore, there are other important molecules with putative functional effects on human health, such as molecules capable of reducing oxidative stress (EC 1.8.1.7, related to glutathione) (41–43) and molecules that can participate in the production of GABA (4-aminobutanoate and l-glutamate) (44, 45). Overall, the screening for enzymatic reactions encoded by the predicted HPCCSTs revealed a unique and significative correlation with enzymatic reaction patterns that support the role of raw milk cheeses as functional foods with a range of impacts on consumer health (Data Set S1).

These data support the drafting of future studies involving additional omics techniques, e.g., metabolomics, that will be pivotal in order to detailing the long-term impact of raw milk cheeses consumption on human health.

### Genomic variability of the raw cheese microbiota across the Italian peninsula.

A comparative genomics analysis was performed to investigate further the genetic microbiome variability that characterizes each PDO cheese and their relationships with the geographical origin and cheese type. In addition, our analyses included metagenomically reconstructed genomes (MAGs). In detail, long reads sequencing was performed for 29 PDO and 10 non-PDO raw milk cheese samples collected across Italy. These cheeses were selected to cover the entire Italian peninsula, prioritizing selecting those cheeses with low species richness to allow efficient metagenomic assembly. Then, long reads were coupled with short reads’ metagenomics data to perform hybrid metagenomics assemblies that led to the reconstruction of draft genomes of the six most prevalent species profiled in raw milk cheeses (Fig. S6). Notably, 71 genomes were selected as they fulfill the average quality standards, i.e., showed >90% of averaged completeness, with <1% contamination and with >94% of average ANI score respect to the species type strain. Thus, corresponding to a number of genomes ranging from 4 to 26 per species that were employed for comparative genomics analyses and pangenomes prediction (Data Set S1) (Fig. S6).

10.1128/msystems.01068-22.6 FIG S6 (a) Bar plot showing the genome completeness score (azure pillar, 0 to 100%) and contamination score (orange pillar, 0 to 100%) of each tested genome. (b) Three different average nucleotide identity matrices, for S. thermophilus*, L. paracasei*, and *L. delbrueckii*, respectively (max score = green, average score = yellow, min score = black). Download FIG S6, TIF file, 1.3 MB. Copyright © 2023 Fontana et al. 2023 Fontana et al. https://creativecommons.org/licenses/by/4.0/ This content is distributed under the terms of the Creative Commons Attribution 4.0 International license.

More than 10 genomes were retrieved from three species out of the six analyzed, i.e., *L. paracasei*, *L. delbrueckii*, and S. thermophilus, and thus their unique gene content, was analyzed (Data Set S1) (Fig. 5).

Subsequently, PGAP pipeline (46) was used to obtain a cluster of orthologous genes (COG) matrix, further processed in order to obtain the presence/absence of all retrieved genes. Then, the recovered matrix of genes presence/absence was used to profile the unique gene content of each genome (Data Set S1).

Additionally, based on the available metadata, Italian regions have been simplified to Islands, North, Central, and South, and then crossed with the average content in unique genes (Fig. 5).

In detail, *L. paracasei* showed an average of unique genes of 117 (standard deviation [SD] of 83.3), 130 (SD of 87.9) and 187 (SD of 54.7) of strains assembled from cheese collected in Island, North, and Center, respectively. Additionally, interpolation of comparative genomics results with other available metadata revealed that strains of the same species reconstructed from different cheeses type also showed high genetic variability, ranging from 40 to 278 unique genes content (Fig. 5). The same type of analysis was also performed for S. thermophilus and *L. delbrueckii*, displaying that the average content of unique genes showed a range from 31 to 215 for S. thermophilus, from 64 to 235 for *L. delbrueckii* and from 40 to 278 for *L. paracasei* (Fig. 5). However, a phylogenetic reconstruction based on the core genes content revealed close evolutionary relationships (Fig. S7).

10.1128/msystems.01068-22.7 FIG S7 Three different phylogenetic trees regarding S. thermophilus*, L. paracasei*, and *L. delbrueckii*, respectively. Type strains are highlighted in red with the exact strain name as label. Download FIG S7, TIF file, 1.4 MB. Copyright © 2023 Fontana et al. 2023 Fontana et al. https://creativecommons.org/licenses/by/4.0/ This content is distributed under the terms of the Creative Commons Attribution 4.0 International license.

These results highlighted a marked genetic variability between different geographical areas and cheese types, supporting once again the role of cheesemaking site-specific NWCs adaptation to unique multifactorial environmental forces, including local raw milk microbiota, through cyclic back-slopping.

**FIG 6:**
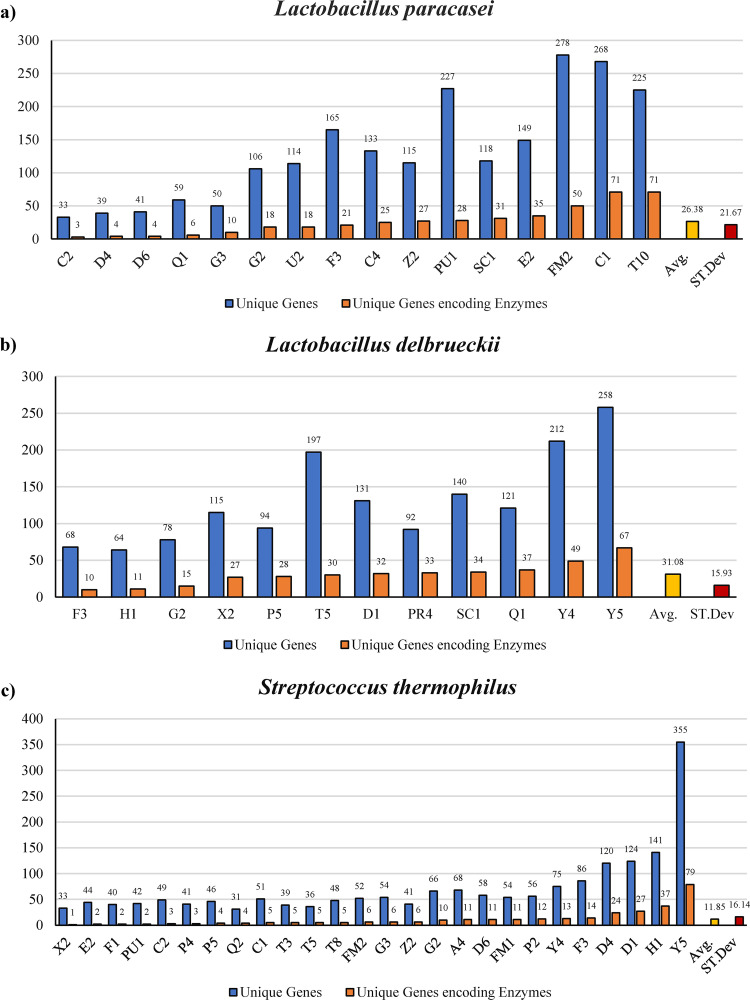
Unique genes content and enzymatic unique potential. (a) Bar plot showing the unique genes content (blue bar) for each different *L. paracasei* genomes tested, along with the unique genes encoding enzymes count (orange bar), the average unique genes encoding enzymes count (yellow bar) and the average standard deviation (red bar). (b) Bar plot showing the unique genes content (blue bar) for each different *L. delbrueckii* genomes tested, along with the unique genes encoding enzymes count (orange bar), the average unique genes encoding enzymes count (yellow bar) and the average standard deviation (red bar). (c) Bar plot showing the unique genes content (blue bar) for each different S. thermophilus genomes tested, along with the unique genes encoding enzymes count (orange bar), the average unique genes encoding enzymes count (yellow bar) and the average standard deviation (red bar).

In this context, screening for enzymatic reactions (against MetaCyc enzyme database) showed that different strains of the same species also possess a unique enzymatic potential (Fig. S8). In detail, *L. paracasei*, *L. delbrueckii*, and S. thermophilus strains showed an average of 26.4 (SD of 21.6), 31.1 (SD of 15.9), and 11.8 (SD of 16.1) unique genes encoding for enzymes, respectively (Data Set S1) (Fig. 6).

10.1128/msystems.01068-22.8 FIG S8 (a) Network correlation of Pearson correlation scores between the different bacteria taxa that arbor inside tested cheeses is reported. Different colors reflect the different modularity cluster (MC) of covariate species found through the use of atlas force 2 algorithm. The diameter of the circles is relative to the prevalence value of each bacterial species between the 103 tested PDO cheese. (b) Detailed summary of modularity cluster compositions (positively covariate bacterial species), with the absolute cells load data regarding the whole modularity cluster compositions. Download FIG S8, TIF file, 1.4 MB. Copyright © 2023 Fontana et al. 2023 Fontana et al. https://creativecommons.org/licenses/by/4.0/ This content is distributed under the terms of the Creative Commons Attribution 4.0 International license.

These strain-unique enzymatic features could be pivotal in the establishment of specific organoleptic features and in the development of bioactive compounds associated with each cheese producer.

Intriguingly, these data support the genetic uniqueness of the strains used to produce different types of PDO cheeses, which could be linked to the use of back-slopping techniques repeated for years as an alternative to commercial microbial starter strains. Therefore, strains naturally present in the NWCs, and originating from the local raw milk microbiota, showed genetic adaptation to the complex set of environmental factor characterizing the production site (external factor as temperature, moisture, milk unique composition and bacterial competition in a semi-isolated system such as the diary factory production system), thus explaining the development of peculiar organoleptic features and potential metabolic profiles that differentiate the final products of each cheesemaker.

The results of this explorative analysis open the avenue of further intriguing future studies aimed at analyzing in detail the functionality of the here described genetic features that characterize different bacterial strains present in cheeses produced in different production sites and subject to different environmental factors.

### Conclusions.

The European PDO quality scheme protects regional raw milk cheese products by standardizing the cheesemaking process based on the know-how of local producers, ensuring that manufacturing is performed in a delimited geographical area using local ingredients. In this framework, increasing interest has recently grown regarding the resident cheese microbiota both for tracing and anti-counterfeit purposes and to disclose microbial communities’ role in organoleptic features development and impact on human consumers.

To investigate these topics, we collected 128 raw milk cheeses across Italy for taxonomic and functional profiling of the resident microbiota. Results revealed how PDO cheeses of the same cheese type denomination but produced from different cheesemaking sites are characterized by unique microbial taxonomical, as well as microbial metabolically and genetic signatures that do not correlate only with their regional origin or cheese type. Instead, there is a vast set of multifactorial modulating factors behind the establishment of unique organoleptic features for each PDO cheese product tested, further linked to the unique composition of manufacturer-specific NWCs that can potentially be associated with the modulation of the final microbiological profiles. Factors that may impact the final taxonomical composition of the cheese products also include the raw milk microbiota used to maintain the NWC and additional environmental factors, such as moisture, temperature, milk composition, and environmental contamination. Thus, the proposal of NWCs as a pivotal factor in the microbial imprinting on final cheese products will need to be confirmed with subsequent *ad hoc* studies.

Notably, these data contrast with the current PDO specification, which relies on the hypothesis of marked regional uniqueness for each specific cheese type denomination. In this way, while PDO certification can lead to the standardization of traditional production processes and guarantee their high-quality standard, it cannot ensure that the same cheese-type PDOs have the same organoleptic characteristics. In this regard, further studies should investigate the potential seasonality effects on the finished product and microbial composition to gain a comprehensive overview of eventual seasonal confounding factors.

Altogether, these functional data underline that a better understanding of the metabolic potential of the microbial communities harbored by raw milk cheeses is pivotal, not only for technological applications, but also for obtaining dairy products with a high-value content of bioactive molecules that could influence the heath of the cheese-consumers.

## MATERIALS AND METHODS

### Sample collection.

A total of 128 Italian cheese samples produced from raw milk were collected from different cheese makers encompassing a large part of the diversity of Italian raw cheese production, considering the main cheese types, different producers, different geographical regions, and both handmade and industrial productive processes. Between one to five samples belonging to different geographical places were collected for each type of Italian PDO raw milk cheese. More details regarding the variety of cheeses have been reported in Table S1. By definition, each sample of cheese is not pasteurized and therefore is not subjected to any heat treatment in order to preserve the bacterial vitality. No precise information regarding temperature of acidification is available because every cheese maker may choose a specific one. Moreover, sample collection focused on cheese certified as PDO, which must respect strict regulations specific for each cheese type that are aimed at preserving artisanal cheesemaking. During December 2019 and January 2020, almost 200 g of each cheese product were kept on ice and shipped to the laboratory under frozen conditions and vacuum packaged, after that they were preserved at −80°C, until they were processed.

### Bacterial DNA extraction and shotgun metagenomics sequencing.

Trying to avoid the rind, a fixed amount of 1 g of cheese belonging to the central portion was homogenized with 9 mL of phosphate-buffered saline (PBS; pH 6.5). Subsequently, 1.5 mL of each resuspended cheese sample was subjected to bacterial DNA extraction using a DNeasy PowerFood microbial kit according to the manufacturer’s instructions (Qiagen, Germany). Then, each cheese sample’s DNA concentration and purity was investigated by employing a Picodrop microtiter Spectrophotometer (Picodrop, Hinxton, UK). The extracted DNA was prepared using the Illumina Nextera XT DNA library preparation kit. Briefly, the DNA samples were enzymatically fragmented to 550 to 650 bp using a BioRuptor machine (Diagenode, Belgium), barcoded, and purified involving the Agencourt AMPure XP DNA purification beads (Beckman Coulter Genomics GmbH, Bernried, Germany). Then, samples were quantified using the fluorometric Qubit quantification system (Life Technologies, USA), loaded on a 2200 TapeStation instrument (Agilent Technologies, USA), and normalized to 4 nM. Sequencing was performed using an Illumina NextSeq 500 sequencer with NextSeq high output v2 kit chemicals (150 cycles) (Illumina Inc., San Diego, CA 92122, USA). All sequencing data were uploaded with BioProject PRJNA865096 and SRA study SRP389312.

### Nanopore sequencing and DNA processing.

Approximately 1 μg of high molecular weight genomic DNA was used to prepare a sequencing library using the Ligation Sequencing Kit (SQK-LSK109) according to the manufacturer’s instructions. For library cleanup, long fragment buffer (LFB) was used to retain DNA fragments. The sequencing library for DNA was prepared in conjunction with the Native barcoding genomic DNA (EXP-NBD104, EXP-NBD114), according to the manufacturer’s instructions. Approximately 50 fmol of the prepared library was loaded onto the R9.4.1 flow cell. Sequencing was performed using the MinION Mk1B sequencing platform. Adaptive sequencing was applied using MinKNOW (21.10.6) software.

### Metagenomics data processing.

Taxonomic profiling of sequenced reads was performed with the METAnnotatorX2 bioinformatics platform (21, 47). In detail, the raw data in fastq format were submitted to quality filtering with removal of reads with an average quality <25. Subsequently, host DNA was removed by reads mapping to the Bos taurus genome. Finally, retained sequences were used as input to perform a MegaBLAST local alignment of reads to preprocessed database, including available genomes of eukaryotes (Fungi and Protists), bacteria, archaea, and viruses. Reads showing a nucleotide identity >94% to the genomes included in the database were classified at the species level, while if a lower percentage identity was detected, they were classified at the genus level as undefined species. These cut-offs are those generally employed for the ANI taxonomic assignment of genomes.

Functional profiling of sequenced reads was performed with the METAnnotatorX2 bioinformatics platform (21, 47) with an updated and manual curated enzymatic database, based on all available RefSeq genomes deposited on NCBI. DIAMOND software was used to assign Enzyme annotation with a MetaCyc updated database through the enzymatic code (EC) unique assignation.

### Evaluation of bacterial cell density by flow cytometry.

For total cell counts, 1 g of each cheese sample was resuspended and homogenized with PBS. Then, 1 mL of the initial homogenized cheese solution was 100,000 times diluted in physiological solution (PBS). Subsequently, 1 mL of the obtained bacterial cell suspension was stained with 1 μL of SYBRGreen I (Thermo Fisher Scientific, USA) (1:100 dilution in dimethyl sulfoxide; Sigma, Germany), vortex-mixed and incubated at 37°C in the dark for at least 15 min before measurement. All count experiments were performed using an Attune NxT flow cytometry (Thermo Fisher Scientific, Waltham, MA, USA) equipped with a blue laser set at 50 mW and tuned at an excitation wavelength of 488 nm. Multiparametric analyses were performed on both scattering signals, i.e., forward scatter (FSC) and side scatter (SSC), while SYBR green I fluorescence was detected on the BL1 530/30 nm optical detector. Cell debris was excluded from acquisition analysis by setting a BL1 threshold. Furthermore, the gated fluorescence events were evaluated on the forward-sideways density plot to exclude remaining background events and to obtain an accurate microbial cell count, as previously described (48). All data were statistically analyzed with the Attune NxT flow cytometry software.

### Statistics and cluster analysis.

HCL analysis was performed on OriginLabPro 2021b (49) with furthest neighbor and Pearson bivariate correlations, a type of analysis that highlight the linear relationships between pairs of continuous variables, ranging in strength and direction from −1 to 1 (50). Eigenvalues scores were retrieved from a Bray-Curtis dissimilarity matrix based on average relative abundance and/or absolute cells load normalized taxonomical profiles of samples, both obtained through the use of Rstudio (51) software. Three- and two-dimensional PCoA representation of eigenvalues scores was made with OriginLabPro 2021b. PERMANOVA statistical analysis was performed on Rstudio (51) software. One-way ANOVA and independent T-test were performed on SPSS software (52) with 1,000 bootstraps. Pearson bivariate analysis was performed with Rstudios software and represented through a correlation Network made with Gephi software using Force Atlas 2 algorithm (53).

### Comparative genomics analysis.

Genome quality assessment was performed manually and through the use of checkM (54) software for completeness and contamination score, fastANI (55) software for the Average Nucleotide Identity between strains of the same species and sourmash (56) software for k-mer based genomes comparison. The pangenome and genes orthologous cluster analysis was performed through PGAP (57) software with –identity 0.5 and –coverage 0.8 as set up. DIAMOND (58) software was used for mapping unique genes protein sequences against a MetaCyc-derived EC database.

### Data availability.

Raw sequences of shotgun data are accessible through SRA under BioProject number PRJNA865096.
