# Supplementary material for: Multifactorial Microvariability of the Italian Raw Milk Cheese Microbiota and Implication for Current Regulatory Scheme
Source: mSystems. 2023 Jan 23;8(1):e01068-22. doi: 10.1128/msystems.01068-22 (PMC9948735; doi:10.1128/msystems.01068-22)
Supplement: TEXT S1 [file msystems.01068-22-s0001.docx]

**Multifactorial dissection of the species-level taxonomic composition across PDO and non-PDO Italian raw milk cheeses.**

In order to understand if there is a region with peculiar cheese microbial-related characteristics, an ANOVA analysis was performed including both non-PDO and PDO cheese in association with their typical region of production. This analysis revealed that five bacterial species significantly differed between regions (Supplementary_Data). Amongst them, one LAB bacterial species widely used as artificial starter in cheese production, i.e., *Lactococcus* *lactis* (1, 2), was significatively different in cheese produced in Tuscany respect to cheese produced from other regions (Supplementary_Data). In detail, non-PDO cheese produced in Tuscany showed an increased abundance of *Lactococcus* *lactis* compared to other cheeses, as expected by the use of this species as a microbial starter culture, which is a common practice in the manufacturing of non-PDO cheeses (One Way ANOVA p-value <0.05) (Supplementary_Data). Consistently, these cheeses also composed an independent cluster in PCoA Analysis (Supplementary Fig. 2, (Point n. 34 – Pecorino Toscano).This PCoA analysis also confirmed the previous observations based on *Lactococcus* *lactis*, i.e. that non-PDO cheeses constitute an independent cluster with respect to PDO cheeses, highlighting the strong relationship (R 21.8%) between the microbial starter cultures and the final cheese microbial composition (PERMANOVA P<0.001 and ANOSIM p-value <0.0001) (Supplementary Fig.2).

**Ecological investigation of microbial correlations in raw milk cheese microbiota.**

To understand the bivariate correlation between dominant and accessory microbial taxa defining the CCSTs, we performed a bivariate correlation analysis between the bacterial species resident in PDO cheeses with an average cell abundance in PDO cheeses > 2.1*10^6 cells. We represented the statistically significant bivariate correlation through a force-driven network (Pearson correlation p-value < 0.05) (Supplementary_Data) (Supplementary Fig.8).

Furthermore, modularity analysis was performed in order to reveal Modularity Clusters (MCs) of species that positively correlate, i.e. their cell counts in PDO cheeses co-variates, for a total of seven final identified MCs (Supplementary_Data) (Supplementary Fig.8).

Intriguingly, these data provided valuable information regarding the ecological forces leading to the stabilization of the final cheese product microbiota such as the network of interactions that the most prevalent species, which dominates in specific CCSTs, establish with a specific range of accessory taxa. Intriguingly, we could not identify any statistically significant negative correlation between selected taxa inside the PDO cheese samples (Supplementary_Data) (Supplementary Fig.8).

Remarkably, three Modularity Clusters (MC) of co-variant species, i.e. MC 1, 2 and 3, showed the presence of at least one taxa identified as dominant in HPCCSTs, which are accompanied by a range of accessory species (Supplementary_Data) (Supplementary Fig.8). This correlation with HCCSTs highlighted how MC 1, 2 and 3 represent the positive correlations between bacterial species that can more frequently be found as dominant taxa in Italian PDO cheeses. In contrast, MCs 4, 5, 6 and 7 were represented by groups of bacterial species that included less than 10 % of the whole PDO raw milk cheeses’ average taxonomic composition expressed as absolute total cells count (Supplementary_Data) (Supplementary Fig.8), thus revealing the existence of low abundance co-variating populations of microbial taxa. Furthermore, this minority MCs showed statistically positive interactions between less prevalent bacterial species that aren’t related to the presence of the dominant taxa defining the HPCCST.

Altogether, collected data allowed us to shed light on the complex ecological network of interactions supporting the development of the cheese microbiota and provided the foundations for developing novel next-generation microbial starters.

1. Lee HW, Kim IS, Kil BJ, Seo E, Park H, Ham JS, Choi YJ, Huh CS. 2020. Investigation of Flavor-Forming Starter Lactococcus lactis subsp. lactis LDTM6802 and Lactococcus lactis subsp. cremoris LDTM6803 in Miniature Gouda-Type Cheeses. J Microbiol Biotechnol 30:1404–1411.

2. Li W, Ren M, Duo L, Li J, Wang S, Sun Y, Li M, Ren W, Hou Q, Yu J, Sun Z, Sun T. 2020. Fermentation Characteristics of Lactococcus lactis subsp. lactis Isolated From Naturally Fermented Dairy Products and Screening of Potential Starter Isolates. Front Microbiol 11:1794.
