# Supplementary figures and images for: Multifactorial Microvariability of the Italian Raw Milk Cheese Microbiota and Implication for Current Regulatory Scheme
Source: mSystems. 2023 Jan 23;8(1):e01068-22. doi: 10.1128/msystems.01068-22 (PMC9948735; doi:10.1128/msystems.01068-22)

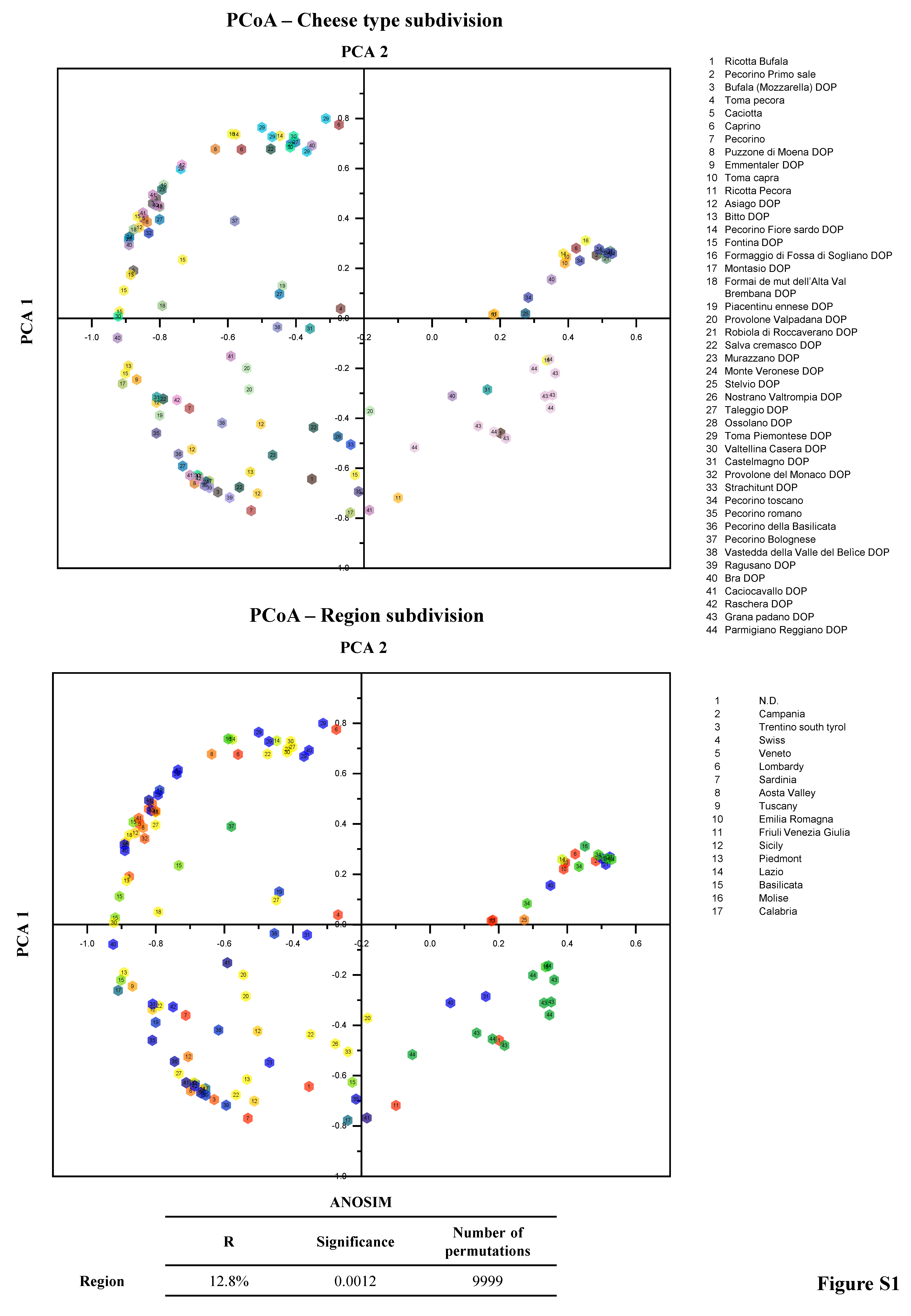

Supplement: FIG S1 [file msystems.01068-22-s0003.tif]

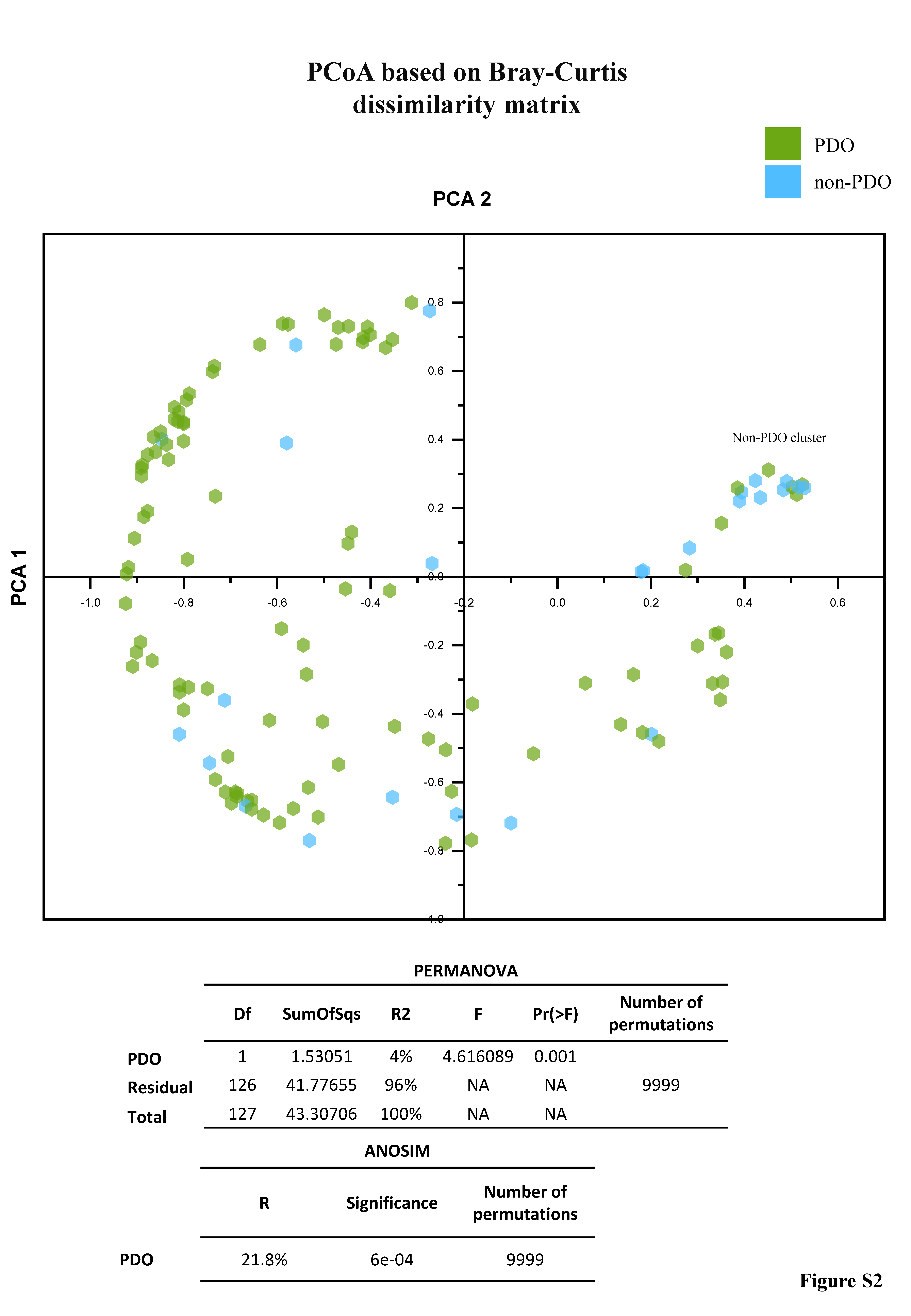

Supplement: FIG S2 [file msystems.01068-22-s0004.tif]

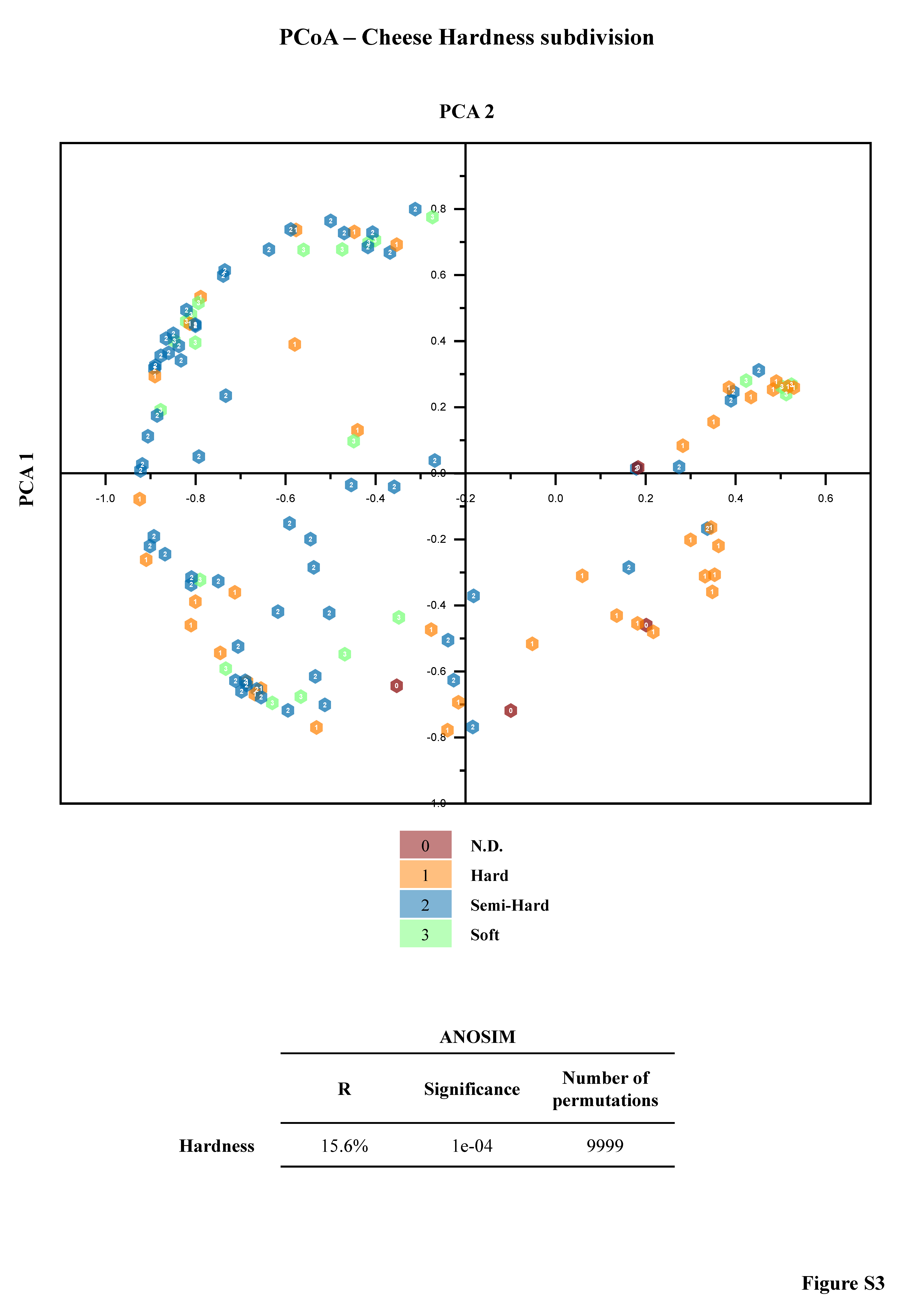

Supplement: FIG S3 [file msystems.01068-22-s0005.tif]

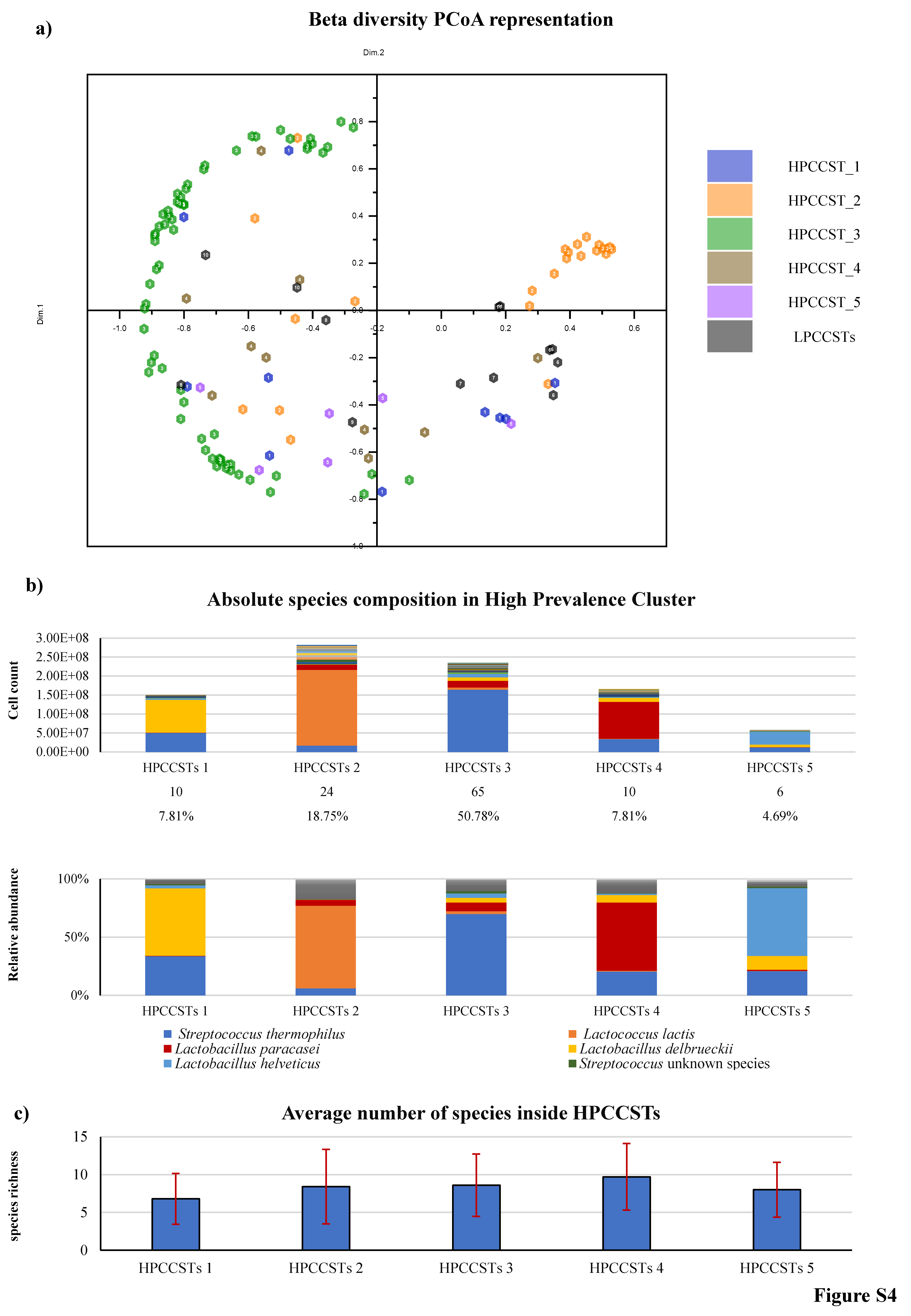

Supplement: FIG S4 [file msystems.01068-22-s0006.tif]

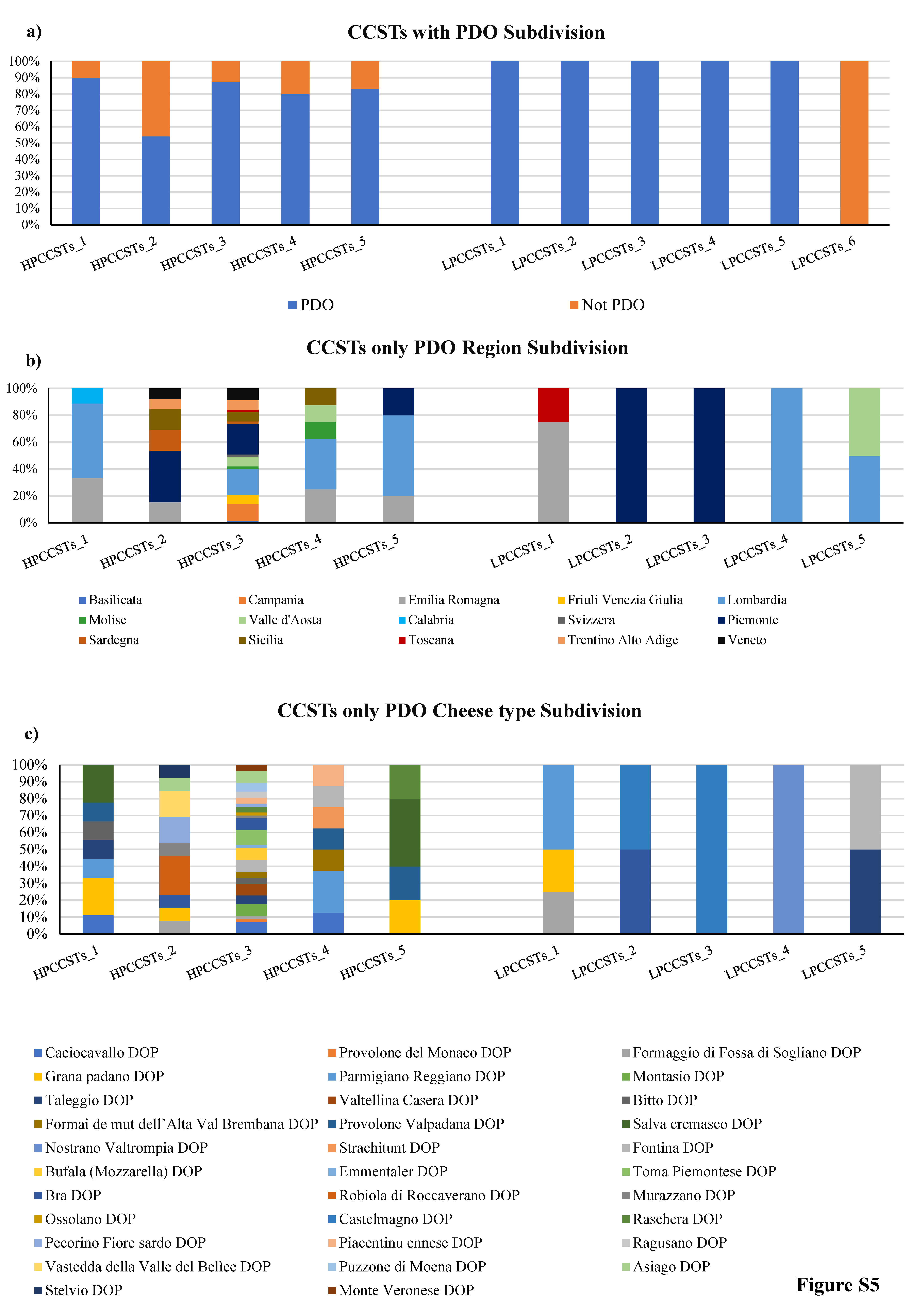

Supplement: FIG S5 [file msystems.01068-22-s0007.tif]

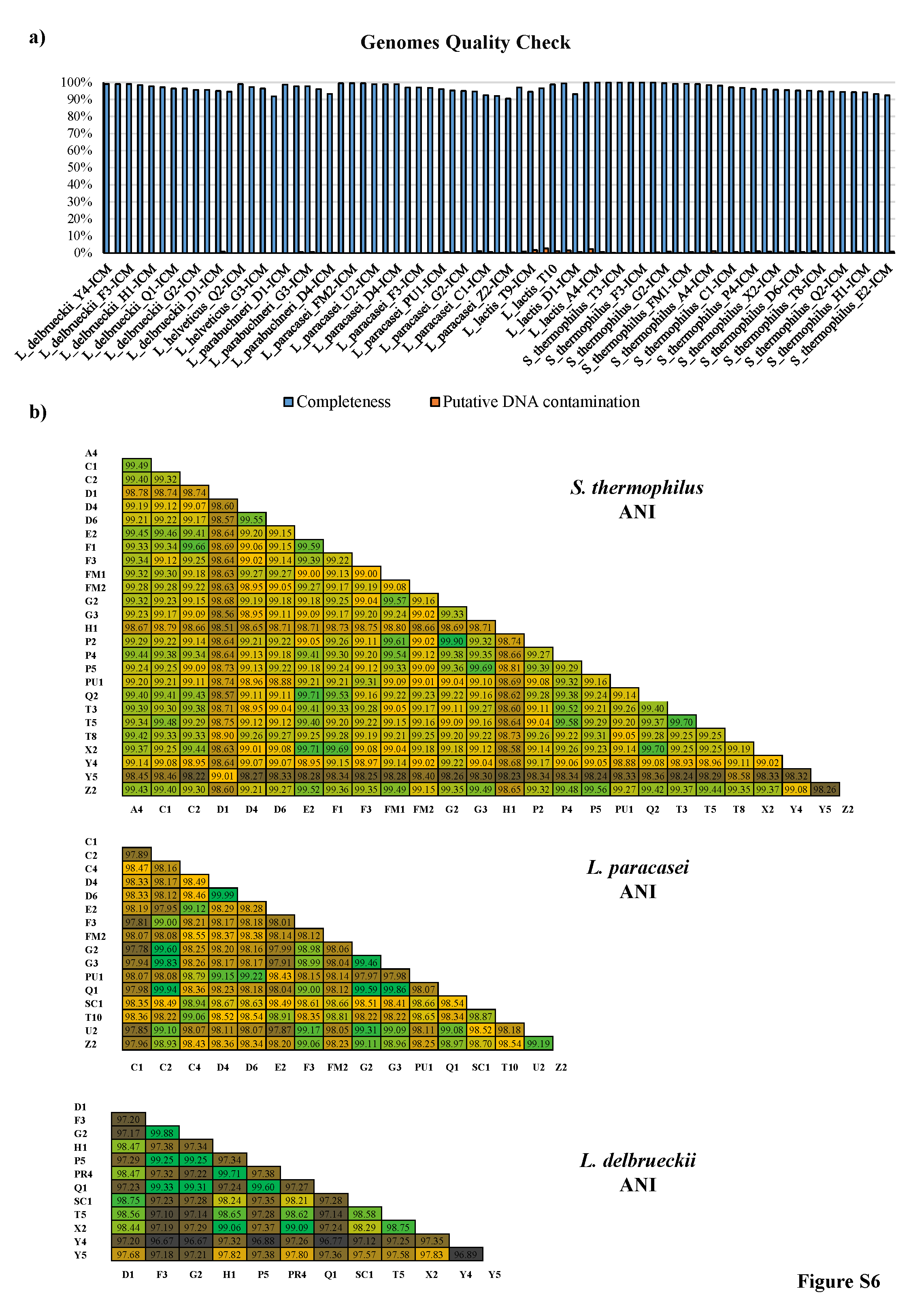

Supplement: FIG S6 [file msystems.01068-22-s0008.tif]

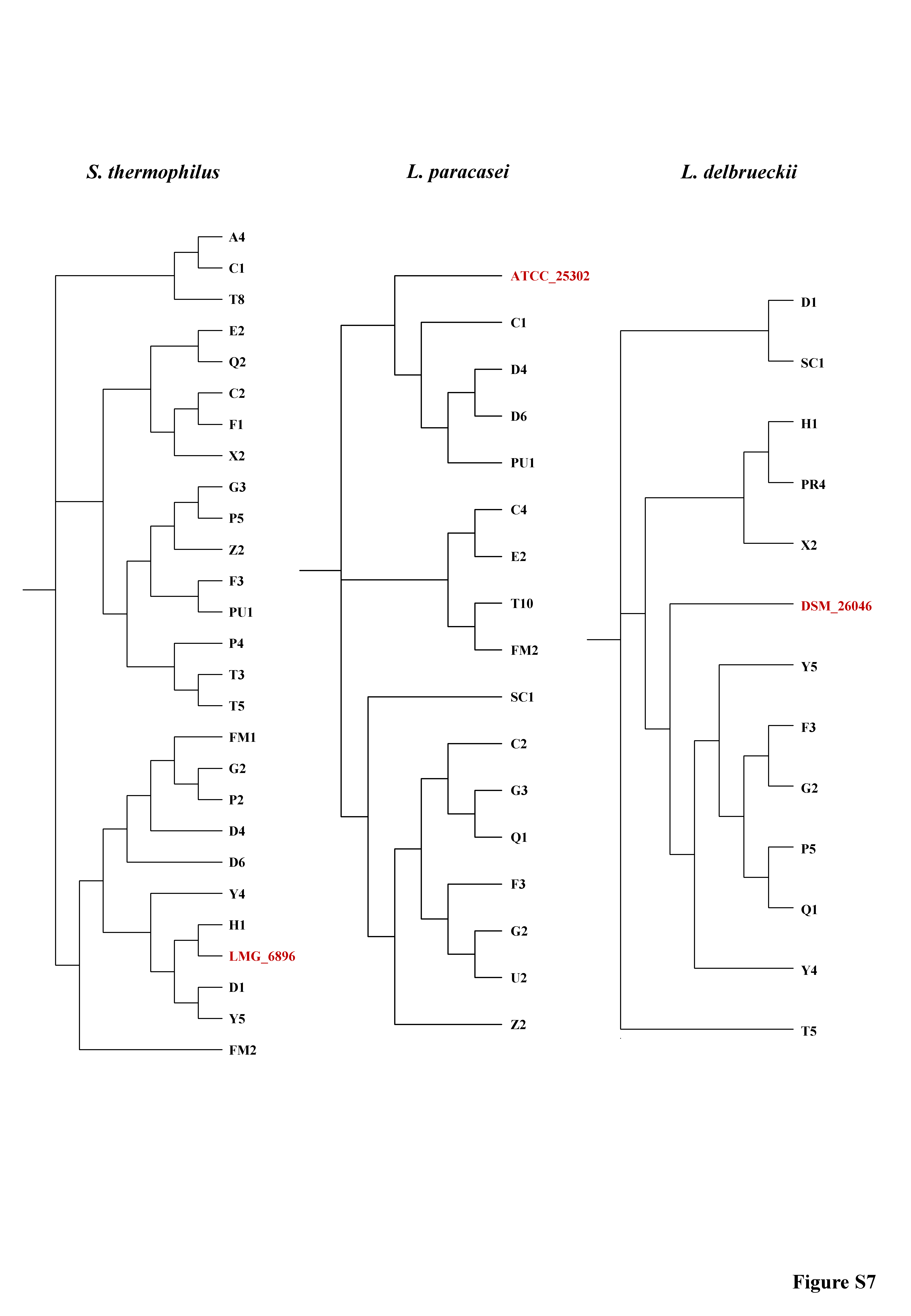

Supplement: FIG S7 [file msystems.01068-22-s0009.tif]

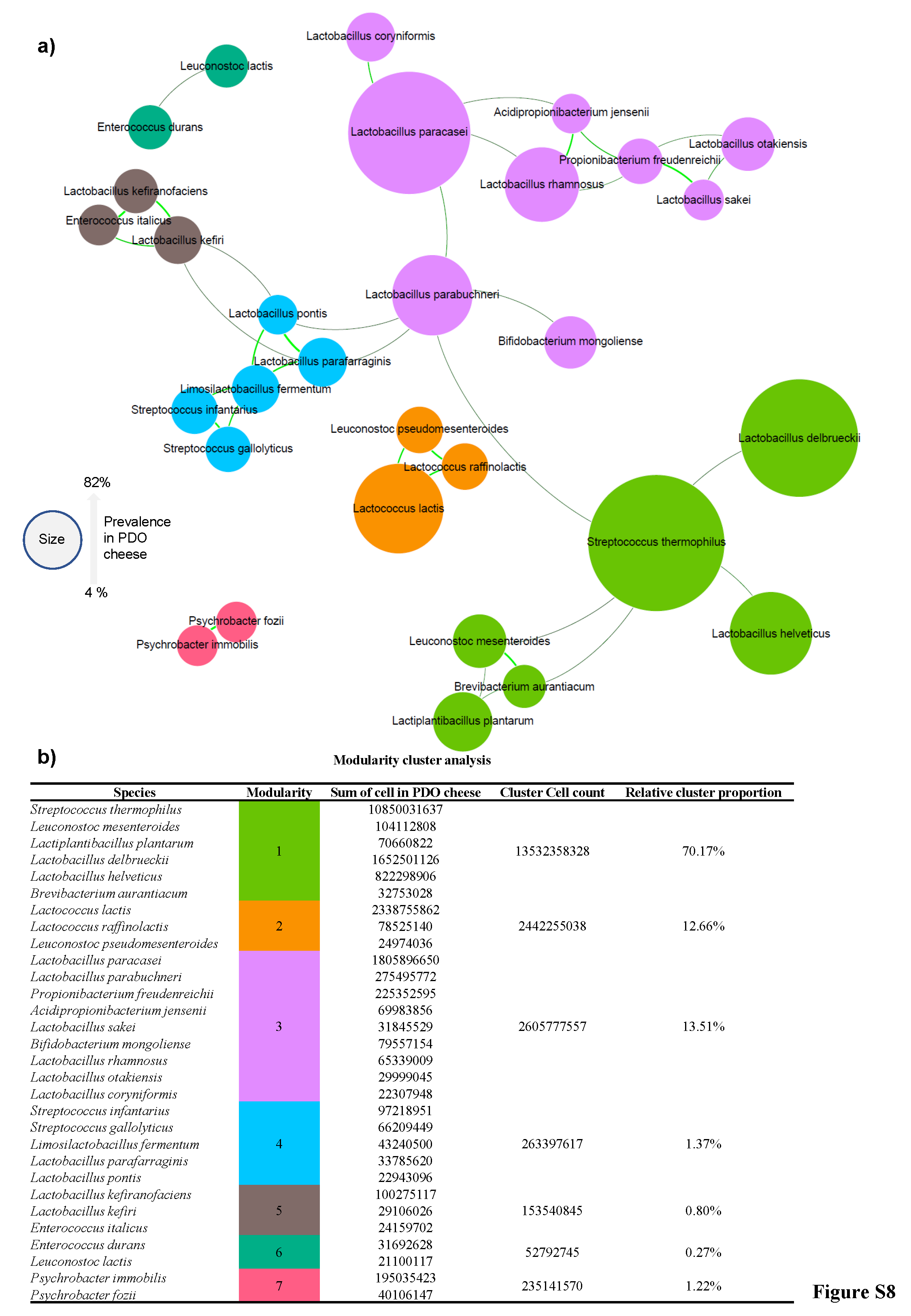

Supplement: FIG S8 [file msystems.01068-22-s0010.tif]
